# The Effect of Fatigue on Postural Control and Biomechanical Characteristic of Lunge in Badminton Players

**DOI:** 10.3390/bioengineering10030301

**Published:** 2023-02-27

**Authors:** Yanyan Du, Yubo Fan

**Affiliations:** 1 Key Laboratory for Biomechanics and Mechanobiology of Ministry of Education, Beijing Advanced Innovation Center for Biomedical Engineering, School of Biological Science and Medical Engineering, and with the School of Engineering Medicine, Beihang University, Beijing 100083, China; 2 Beijing Key Laboratory of Sports Function Assessment and Technical Analysis, School of Kinesiology and Health, Capital University of Physical Education and Sports, Beijing 100191, China

**Keywords:** balance, COP, fatigue, badminton, kinematics, kinetics

## Abstract

This study investigated the effects of fatigue on postural control and biomechanical characteristic of lunge. A total of twelve healthy male collegiate badminton players (21.1 ± 2.2 years; 180.8 ± 4.0 cm; 72.5 ± 8.4 kg; 8.9 ± 3.5 years of experience) performed repeating lunges until exhausted. Postural stability was evaluated through a single-leg balance test using the dominant lower limb on a pressure plate with eyes opened (EO) and eyes closed (EC). The center of pressure (CoP) sway in the entire plantar and sub-regions of the plantar was measured. Kinematic and kinetic data of lunge motion were collected. The postural control was impaired after fatigue. In plantar sub-regions, the area, displacement and distance in the medial–lateral (ML) and anterior–posterior directions of CoP increased significantly (*p* < 0.05), especially the distance in ML. The medial region of the forefoot is the most sensitive to fatigue. Compared to pre-fatigue, participants experienced a significantly longer phase of pre-drive-off (*p* < 0.01), less peak moment and peak power of the knee and hip for drive-off (*p* < 0.01) and less peak moment of the ankle during braking phase (*p* < 0.05). These findings indicate that, within the setting of this investigation, the different responses to fatigue for CoP sway in plantar sub-regions and the consistency between postural control and biomechanical characteristic of lunge may be beneficial for developing and monitoring a training plan.

## 1. Introduction

In badminton, balance is one of the key factors for an effective shot. Previous studies have demonstrated that muscle fatigue can reduce the maximum voluntary muscle force and work capacity [1] and impair the proprioceptive system, which in turn can contribute to a decrease in joint stability, and can alter movement control [2] and impair the postural control [3,4,5]. Additionally, these performance changes under fatigue might increase the risk of injury [6]. An epidemiological study reported that badminton is one of the games with a high injury rate [7]. Overuse injury caused by high-intensity training and repetitive movement is the main type of injury [8,9]. However, the responses of fatigue on balance in badminton are unknown, and information is needed to provide insight about the postural control changes which occur after fatigue.

A wide variety of fatigue protocols were used in fatigue studies, including isometric [5], treadmill running, specific fatigue [3,10], simulating match [11] and so forth. These are generally categorized as either general fatigue or peripheral nerve fatigue. Additionally, it is verified that the fatigue protocols did not uniformly produce alterations in lower-limb neuromuscular factors related to the high risk of injuries, such as an ACL injury [12]. Density and duration of training [13], anatomical position to move [14] and duration of concentric/eccentric movement would all lead to distinct muscle activation and neuromuscular fatigue responses. Consequently, fatigue protocol is the key part for the interpretation of results. Considering that fatigue effects occur cumulatively throughout a practice or a game and the entire athletic season [12,15], some protocols attempt to simulate realistic match [11] or game situations focusing on a specific relevant skill [16] to provide support for training. In badminton, the lunging action is a basic and important footwork [17]. When repeating lunges until fatigued, the activity of vastus lateralis and biceps femoris showed significant change [18]. Joint stiffness in the knees during a forward lunge task increased after fatigue due to the repeated forward lunges by badminton players. This would then influence their performance and increase the injury risk [19]. A study has reported that the dominant (leading) limb, acting as a generator of vertical force during a lunge, may contribute to the development of muscular imbalances, which may ultimately contribute to the development of an overuse injury [20]. Compared to other directions, the left-forward and right-forward lunge directions were associated with higher plantar loading at the heel and toe regions. However, few studies pay attention to the changes of postural control and lunge performance after fatigue in badminton.

Generally, the sway of center of pressure (CoP) standing on a force plate system [21] or balance system [22] is used to assess the postural sway. However, the pressure plate system was confirmed to provide reliable and valid measures of static standing balance [9]. In addition, significant differences were found in the pressure distribution of plantar sub-regions [15,23,24] during the stance phase of a lunge. These differences in the postural, caused by fatigue, may also induce a different distribution of plantar pressure. However, to the best of the authors’ knowledge, there is no research that has studied and discussed this.

Consequently, the aim of the present study was to investigate the effect of fatigue caused by repeating lunges on the postural control and the biomechanical characteristics of lunges. To reach the objective, repeating forward lunges until exhaustion was proposed as fatigue protocol. The pressure plate was used to access the sway of CoP in whole and sub-regions of the dominant plantar. Additionally, the following hypotheses were formulated: (1) postural sway would increase after the fatigue protocol, (2) different sub-regions of the plantar would have different responses to fatigue and (3) the kinematics and kinetics of the lunges would be affected by fatigue.

## 2. Materials and Methods

A total of twelve healthy male collegiate badminton athletes (21.1 ± 2.2 years; 180.8 ± 4.0 cm; 72.5 ± 8.4 kg; 8.9 ± 3.5 years of experience; 2–4 h badminton training per day) were recruited by badminton coaches from two universities for the study. All participants were free from musculoskeletal or neurological conditions, and without lower limb injuries in the last three months.

Prior to participating in the study, all participants were informed of the experimental procedures and potential risks, and then they signed an informed consent document. Additionally, a questionnaire about their anthropometrics, healthy status, injuries history, training plan and physical activity levels were filled out. They all wore badminton footwear of the same brand and series, avoiding the effect of footwear. The experiment was approved by the Ethics committee of Beihang University.

Prior to the test, participants took 15 min to warm up and become familiar with the footwork and court. In addition, they were sure to stop when they experienced discomfort at any time. Then, we started the test. Heart rate (HR), blood lactate (BL) and a Borg 6–20 rating of perceived exertion (RPE) were assessed at baseline (0), immediately (T0) after exhaustion and at the 6th (T6) and the 9th minute (T9) after the protocol, accounting for the time for the testing of HR, BL and RPE. Participants completed the single-leg balance test at baseline and immediately following the HR, BL and RPE testing after the fatigue protocol. Kinematics and kinetics of the lunge were recorded by motion capture system and force platform. After fatigue, all tests were completed within 10 min.

**Figure 1:**
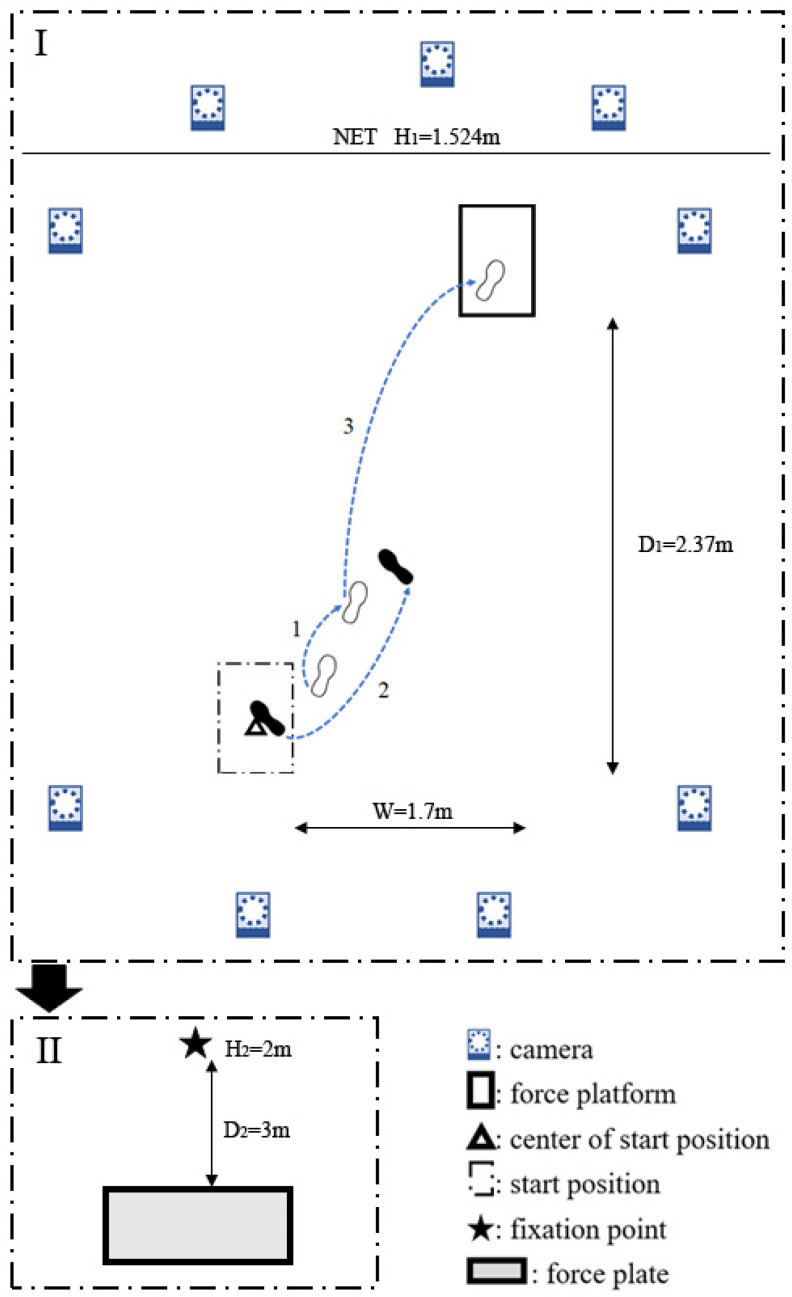
Illustration of footwork and testing process. The badminton athletes lunged and followed the footwork shown in I, and repeated until exhausted. Then, they took II, the poster sway test, standing on a plantar pressure plate using their dominant leg. In the illustration of lunge footwork, the right leg is dominant. The open foot marks represent the foot placements of the right foot, whereas the solid foot marks represent the foot placements of the left foot. The numbers represent the step sequences.

Repeating forward lunge until exhaustion was proposed as the fatigue protocol [25]. The protocol was elaborated to be conducted in a simulated badminton court. One forward lunge was defined as starting from the starting position, lunging with a sliding step to the force platform, kicking the shuttlecock, and moving back to the starting position (details are illustrated in part I of Figure 1). An 80–90% maximus range of lunge was repeated once for approximately 2.8 s until they were exhausted. A metronome was used to establish the rate. Participants were instructed to follow the rhythm, and stop when they could not follow the rhythm for 10 s.

HR (beats per minute, bpm) was assessed by the Polar heart rate sensor H1 (Polar Electro, Kempele, Finland) throughout the test processing. BL (mmol/L) was measured by a portable blood lactate meter (SensLab GmbH, h/p/cosmos sirius^®^, Leipzig, Germany). Additionally, RPE was recorded. Fatigue was decisively judged by the values of maximum HR [18], BL and RPE [26].

Here, maximum HR (HRmax) was calculated as 220 minus age (year). The criteria included (1) HR being greater than or equal to HRmax, (2) BL being greater than or equal to 8 mmol/L and (3) the value of RPE being greater than or equal to 18. Once two of the criteria were fitted, the participants were exhausted. The testing was completed, and it was a valid test. Additionally, the testing was interrupted when one participant was too fatigued to continue the testing, even if no criterion was reached.

Participants stood upright on a pressure plate (40 × 100 cm, 1 m-3D; footscan^®^ system, RSscan International, Olen, Belgium) in a one-leg posture with the dominant limb, arms by their side. They were instructed to stand as quietly as they could with eyes opened (EO), looking at a target positioned 3 m away (details are illustrated in part II of Figure 1), and with eyes closed (EC). Participants lifted the non-dominant leg upon an auditory signal. A primary investigator performed all balance tests. The balance module of pressure plate system was set with a 5 s delay after the auditory signal and to record for 5 s (100 Hz, 128 Lines/plate).

**Figure 2:**
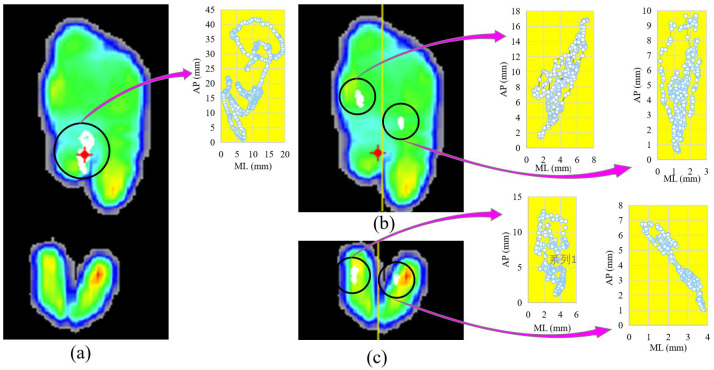
One example of the center of pressure (CoP) of planter and its sub-regions while standing on the dominant leg with eyes opened, before fatigue. (**a**) The maximum-pressure image and the sway of CoP; (**b**) the top of plantar is divided into two sub-regions along the yellow line, and the sway of CoP; (**c**) the bottom of plantar is divided to two sub-regions along the yellow line, and the sway of CoP of plantar and sub-regions.

Plantar was divided into top (forefoot) and bottom (rearfoot) regions (T, B), and then further divided into medial and lateral regions (TM, TL, BM, BL, illustrated in Figure 2), using the software of pressure plate system. Threshold level for pressure was 10 N. The center of pressure (CoP) sway of entire and sub-regions were exported from the software. For each trial, the following variables were calculated: ellipse area containing 95% of the CoP data points (Area), sway displacement (Dis) and distance in medial–lateral (ML) and anterior–posterior (AP) regions.

A nine-camera motion capture system (Qualysis, Göteborg, Sweden) sampling at 200 Hz and one Kistler force platform (Kistler, 9286 A, Kistler Instrument AG, Winterthur, Switzerland) sampling at 1000 Hz were used to collect the ground reaction forces (GRF) and kinematic data, simultaneously. According to the CAST lower-leg model [27], reflective markers (18 mm diameter) were firmly placed over the hip and lower legs. The kinematic and kinetic data of lunge motion were collected.

The kinematic and force data were obtained by the optical motion capture system and then exported and saved as c3d files. Then, for the dominant lower limb, the hip, knee, and ankle joint angles, moments, power and ground reaction force (GRF) during stance phase of lunge were calculated using visual 3D software (V5, C-Motion, Bethesda, MD, USA). The raw kinematic data were filtered with a low-pass (Butterworth) filter, with a frequency of 20 Hz [10]. The threshold of the vertical ground reaction force (vGRF) data was set at 10 N.

The stance phase, from initial contact (heel strike) to final lift-off from the force-plate by the dominant limb, was determined by the vGRF value. Based on previous studies [20,25], the stance phase was divided into five phases: I (0~initial impact peak (PF1)), II (PF1~secondary impact peak (PF2)), III (PF2~peak angle of knee flexion (PAK)), IV (PAK~third peak during drive-off (PF3)) and V(PF3~end). Based on the previous literature linked to the lunge in badminton [15,20,25], we analyzed the impact peak, duration of five sub-stance phases, hip, knee, and ankle joint initial contact angles, durations to peak angle, ranges of motion (RoM), peak angles, moments and power in the sagittal plane.

Results are reported as mean ± standard deviation (SD). All variables in this study were examined for normality using a Shapiro–Wilk test prior to statistics analysis. The force, joint moment and power were normalized to body mass. One-way Repeated-Measures ANOVA was used for the analysis of the influence of fatigue on the related parameters, including fatigue parameters (HR, BL and RPE) between the baseline (0), T0, T6 and T9, the postural sway variables and biomechanical data. Paired *t*-tests were performed to identify differences. All statistical procedures were performed with IBM SPSS Statistics for Window (Version 25.0; IBM Corp., NY, USA). Additionally, a statistical significance level was accepted at 0.05. Effect size (Cohen’s *d*) was computed for the *t*-test. Small, middle and large effect sizes were 0.2 ≤ *d* < 0.5, 0.5 ≤ *d* < 0.8 and *d* > 0.8, respectively.

## 3. Results

**Figure 3:**
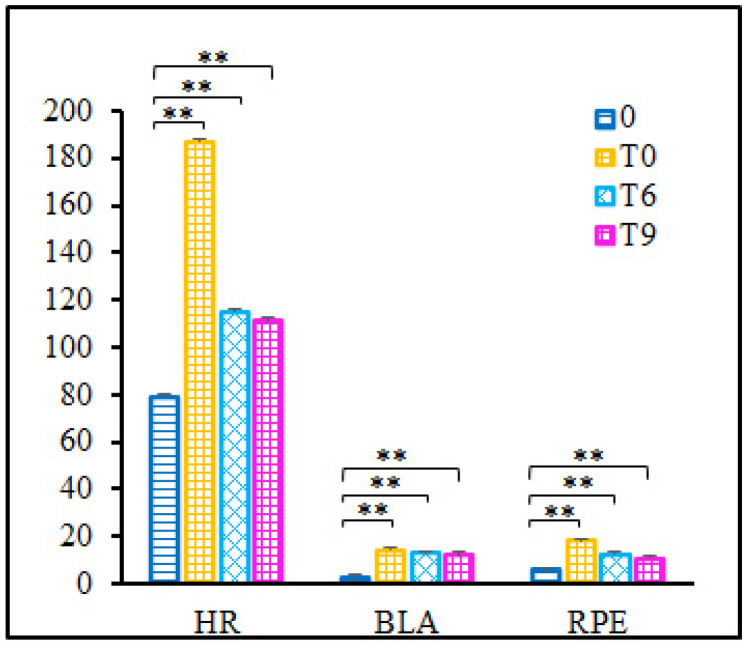
Rate of heart (HR) (beats per minute, bpm), blood lactate (BLA) (mmol/L) and Borg 6–20 rating of perceived exertion (RPE). 0—baseline, before fatigue protocol; T0—immediately after fatigue protocol; T6—the 6th minute after fatigue protocol; T9—the 9th minute after fatigue protocol. Note: ** indicates the significance level *p* < 0.001.

For the HR, BL and RPE, the mean and SD values are illustrated in Figure 3. Significant increases were observed for HR (*p* < 0.001), BL (*p* < 0.001) and RPE (*p* < 0.001). Although these values decreased at T6 and T9, they showed significant differences from the baseline (*p* < 0.01).

**Figure 4:**
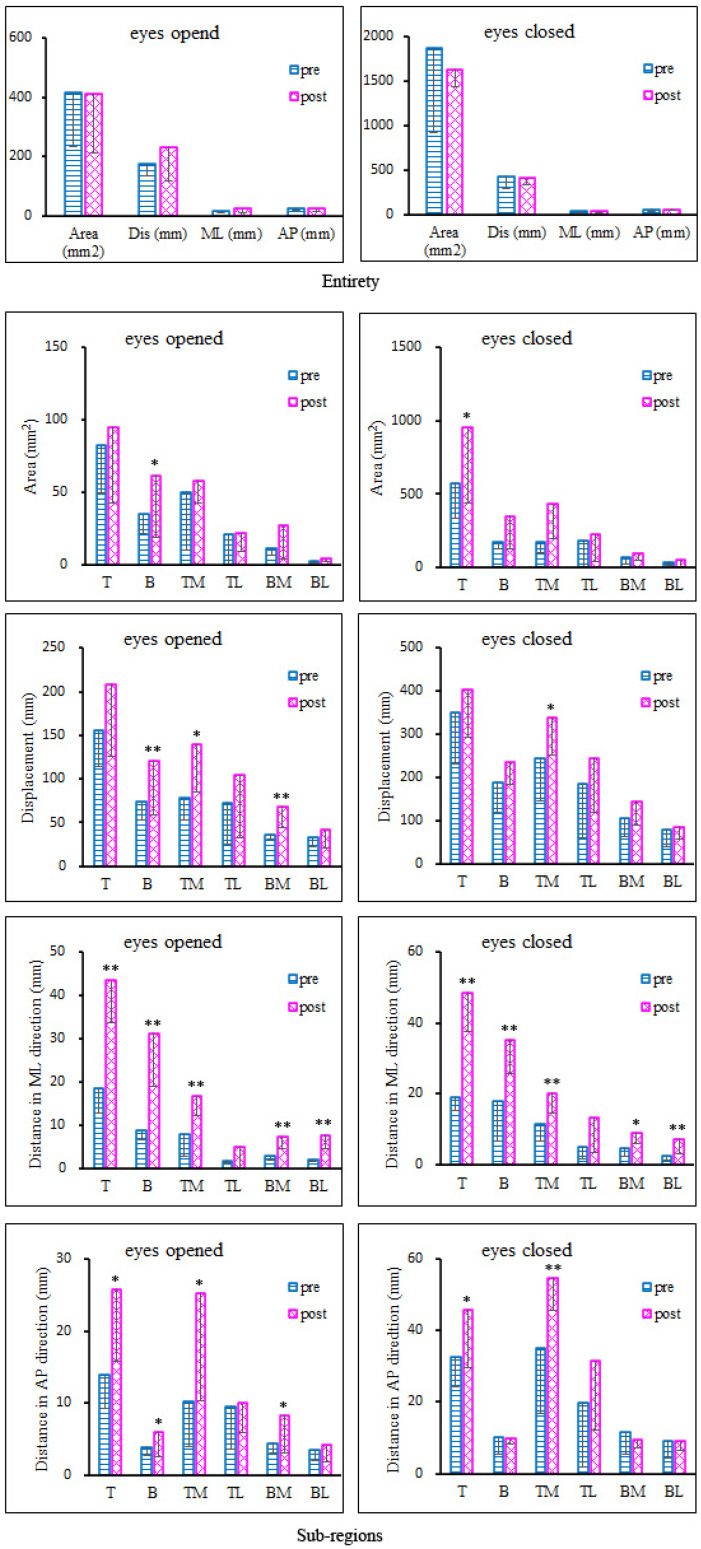
Standing on the dominant leg with eyes opened and closed. Plantar was divided into the following six sub-regions: top (T, forefoot), bottom (B, rearfoot), medial and lateral of the top (TM, TL) and medial and lateral of the bottom (BM, BL). The area (mm^2^), displacement (mm), sway distance in medial–lateral (ML) and anterior–posterior (AP) regions of postural sway are shown for the entirety and sub-regions using the pre- and post-fatigue protocols. * indicates the significance level *p* < 0.05; ** indicates the significance level *p* < 0.01.

For the variables of postural sway in the entire region, Figure 4 (Entirety) shows that with either eyes opened or closed, all variables had no significant change after the fatigue protocol.

For sub-regions, both with EO and EC, almost all of the postural variables for all sub-regions increased after the fatigue protocol (illustrated in Figure 4, Sub-regions). With EO, area of rearfoot (*p* = 0.04), displacement of rearfoot (*p* = 0.006), medial forefoot (*p* = 0.046), medial rearfoot (*p* = 0.003), distance in ML of forefoot (*p* < 0.001), rearfoot (*p* < 0.001), medial forefoot (*p* = 0.001), medial rearfoot (*p* < 0.001), lateral rearfoot (*p* < 0.001), distance in AP of forefoot (*p* = 0.014), rearfoot (*p* = 0.035), medial forefoot (*p* = 0.031) and medial rearfoot (*p* = 0.032) increased significantly within 10 min after the protocol (Figure 4). With EC, variables also increased significantly, specifically, area of forefoot (*p* = 0.05), displacement of medial forefoot (*p* = 0.035), distance in ML of forefoot (*p* < 0.001), rearfoot (*p* < 0.001), medial forefoot (*p* = 0.002), lateral forefoot (*p* = 0.017), medial rearfoot (*p* = 0.014), lateral rearfoot (*p* = 0.006), distance in AP of forefoot (*p* = 0.029) and medial forefoot (*p* = 0.004) (Figure 4).

Table 1 shows that the stance phase was divided into five sub-phases by the time of peak force (initial impact, secondary impact and drive-off) and peak angle (ankle plantar flexion and knee flexion). For pre- and post-fatigue, there were statistically significant differences in the time of drive-off impact peak (*p* = 0.005, *d* = 1.410); the duration of phases III, IV and V (*p* = 0.041, 0.002, 0.005; *d* = 0.883, 1.701, 1.410, respectively); time of peak angle (T%) for ankle and knee in sagittal plane (*p* = 0.001, 0.009; *d* = 1.972, 1.270, respectively).

The initial contact angle of the knee and the hip, the peak knee flexion and the knee RoM in sagittal plane decreased significantly after fatigue (*p* = 0.004, 0.044, 0.008, 0.048; *d* = 1.476, 0.868, 1.296, 0.848, respectively) (Table 2).

For PF1, PF2 and PF3, Table 3 shows no differences between pre- and post-fatigue. Peak joint moment and power in sub-phases were calculated. The peak joint moment in the sagittal plane (knee—I, II and III: *p* = 0.004, *d* = 1.394, IV and V: *p* = 0.011, *d* = 1.445; hip—I, II and III: *p* = 0.027, *d* = 0.893, IV and V: *p* = 0.009, *d* = 1.230) and in the frontal plane (ankle—II: *p* = 0.026, *d* = 0.996, III: *p* = 0.033, *d* = 0.934), and joint power in the sagittal plane (knee—I, II and III: *p* = 0.009, *d* = 1.268, IV and V: *p* = 0.000, *d* = 2.325; hip—IV and V: *p* = 0.014, *d* = 1.157) decreased after fatigue.

## 4. Discussion

The purpose of this study was to investigate the effect of fatigue on postural control and biomechanical characteristic of lunges. The results demonstrated that: (i) the postural control was impaired within 10 min after fatigue, (ii) special postural variables and plantar sub-regions were more sensitive to fatigue, and (iii) the changes of kinematics and kinetics of lunges were consistent with postural impairment.

Contrary to studies that induced fatigue using repetitive isokinetic or isometric contractions, the current study used repeated forward lunging as the fatigue protocol. This way, the musculoskeletal system, load and angular velocity of the lower extremity joints were consistent with a badminton game. To a certain degree, this can better represent the state of fatigue in daily training and matches. Results showed that HR (HR/HRmax: 87.8~99.6%, mean ± SD: 93.5 ± 3.3%), BL (8.8~18.2 mmol/L, mean ± SD: 13.8 ± 2.7 mmol/L) and RPE (14~20, almost all data were greater than or equal to 18, except for one that was 14 and another that was 17; mean ± SD: 18.1 ± 1.5) increased immediately after the fatigue protocol. In combination with the results of the statistical analysis, it is reasonable to consider that the fatigue protocol induced fatigue in these participants.

Considering the effect of fatigue recovery, we measured the HR, BL and RPE at the 6th and 9th minutes after the fatigue protocol and performed the tests within 10 min. Despite HR, BL and RPE decreasing and being less than the criteria at T6 and T9, the BL was greater than 8 mmol/L at T9. Additionally, results of the statistical analysis also showed a significant increase at T6 and T9 for HR, BL and RPE. Consequently, we considered that the participants were fatigue during the test processing.

The postural control was impaired after the fatigue protocol. It is consistent with the conclusion that fatigue would minimize the ability to keep balance [3,4,5] and increase postural sway. However, a study [22] that adopted the Bosco protocol found that muscle fatigue of the lower limbs would affect vertical jump (VJ) performance, with no effect on balance. The discrepancy with the current study may indicate that the method to test the postural control differs. While VJ testing is the most common tool to explore lower-body power and strength in all sports [22], it can not follow the different movement strategies with similar total power output. Another study [19] also took repeating forward lunges as the fatigue protocol and showed changes in movement control and strategy during the lunge tasks, with no significant differences between pre- and post-fatigue for the Y-balance test. Such inconformity may be caused by the sports level of the participants [7] and the insensitivity of those clinical scores to the changes of performance.

It is worth noting that the effects of fatigue were not in the postural variables of the entire region, but those of plantar sub-regions. The rearfoot and forefoot were the sensitive regions for EO (area: *p* = 0.05; distance in ML: *p* < 0.001; distance in AP: *p* = 0.029) and EC (area: *p* = 0.04; displacement: *p* = 0.006; distance in ML: *p* < 0.001; distance in AP: *p* = 0.035), respectively. The medial of the forefoot is the most sensitive region to the fatigue protocol, with significant increase in displacement (EO: *p* = 0.046; EC: *p* = 0.035), distance in ML (EO: *p* = 0.001; EC: *p* = 0.002) and AP (EO: *p* = 0.031; EC: *p* = 0.004). As an asymmetrical movement, the dominant leg needs to complete the landing to the ground with the heel, lunging, supporting, braking and taking off. There are different loads in different plantar sub-regions [15,23,24]. It is well known that plantar cutaneous receptors help us balance and stand upright. The sensory feedback of the big toe and forefoot play an important role in the balance control of single-leg standing [28]. However, without data, it cannot verify whether the changes of postural sway were affected by the plantar cutaneous receptors. A study has confirmed that adding body load modified the vibratory sensation of the foot’s sole, and it also significantly increased the CoP surface and lateral deviation [29]. In addition, the decreased ability of the musculoskeletal system caused by fatigue may be another reason for an increase of postural sway. In this study, peak powers of the knee (flexion and extension) and hip (extension) all decreased significantly. Similar results were found in a previous study [30]. Consequently, it may indicate that fatigue is only one of the factors contributing to the posture disorder.

Furthermore, among these variables, distance in ML is the most sensitive one to fatigue. During lunging, the gastrocnemius is the major one. A study has confirmed that the fatigue of gastrocnemius muscle had more obvious influence on the balance stability of the ML directions [31]. However, another study reported that the impairments in postural control were more evident in the AP direction when the plantar flexor and dorsiflexor muscles were affected [32]. Further work should focus on the function of these muscles.

Contrary to the negative effect of fatigue [14], proper neuromuscular training can improve the awareness of lower-limb joint and posture control [33]. Thus, studies of the influence of fatigue on the postural ability may provide a reference for reasonable and scientific training.

No differences were found for peak impact for the initial, secondary and drive-off phases. However, the timing of these variables was earlier than pre-fatigue, which can be explained by the decrease in postural control. After fatigue, the duration of III, between peak knee flexion and peak drive-off impact (PF2-PAK), increased significantly (pre: 27.3 ± 10.7 T%, post: 43.4 ± 15.2 T%, *p* = 0.002). This means that players spent more time preparing for drive-off. During this sub-phase, larger knee RoM, combined with the smaller knee joint moment in the sagittal plane, stands for the decrease of muscle strength. In addition, more attention should be paid to the angle of the ankle in frontal region. Smaller ankle eversion during stance might be a potential contributor to the injury risk of the ankle [8,15]. Except for the decrease in ankle eversion after fatigue, smaller joint moment of ankle in frontal (*p* = 0.033) is found, which means the induced ability of evertors around ankle. This may be used to explain why CoP sway in ML is more sensitive to fatigue. In badminton, there are higher rates of injury in the ankle and knee. Overuse injury caused by high-intensity training and repetitive movement is the main type of injury [8,9]. Lunging is an important footwork, with high-intensity use during training and competition [17]. During stance phase of the lunge, smaller knee joint moment in the sagittal (*p* < 0.05) may link to induced extensor muscle strength around the knee after fatigue. In addition, it is worth considering the changes in the hip. Before heel contact, sufficient flexion in the hip is important for a lunge. However, the smaller flexion angle of the hip at initial contact (*p* = 0.044) and hip joint moment in the sagittal plane are reported after fatigue. Furthermore, during drive-off, less power in the knee and hip in the sagittal plane may illustrate the decrease in control of the postural region. These suggest that specific muscles around the lower leg joints should be improved to maintain lunge performance, especially post-fatigue.

Considering the findings of this study, it did have a few limitations that should be considered when interpreting the results. Firstly, in the present study, only the dominant leg was tested for the single-leg balance test. Fatigue responses of non-dominant leg [34,35] and dynamic balance should be considered in further studies. Secondly, five seconds may give limited information, although a five-second delay was set for the postural test and one primary investigator performed all the tests. Further work should be conducted so as to determine the duration of the influence of fatigue recovery on postural control. Finally, all tests were performed in a simulated badminton court, and the participants were only college-aged male badminton players, with a limited sample size. Players of different genders, ages and sports levels may show different performance skills in badminton and may have different fatigue responses. A future study should take consideration of these differences.

## 5. Conclusions

In summary, the fatigue protocol had a significantly negative effect on postural control and biomechanical characteristic of lunge. The changes in biomechanical characteristic were consistent with the impairment of postural control. CoP sway of plantar sub-regions assessed by pressure plate system showed a significant response to fatigue. It provided us with the possibility of using postural sway data to monitor the state of motion, and to make an appropriate and scientific training plan to improve control ability and reduce the incidence of injury. On the other hand, future studies should extend the postural test time, combined with the EMG and dynamic balance test for a better understanding. Notice that avoiding the effect of fatigue recovery is important.

## Figures and Tables

**Table 1:** Time of peak force (T%), sub-phases (T%) and time of peak angle (T%) (mean ± SD).

	Pre-Fatigue	Post-Fatigue	*t*	*p*	Cohen’s *d*
Time of peak force (T%)					
Initial impact	3.9 (0.6)	3.8 (0.7)	0.4	0.685	0.150
Secondary impact	15 (3.2)	12.6 (3.3)	2.2	0.065	0.774
Drive-off impact	73.1 (5.6)	77 (6.7)	−4	0.005 *	1.410
Phases (T%)					
I (0-PF1)	3.9 (0.6)	3.8 (0.7)	0.4	0.685	0.150
II (PF1-PF2)	11.1 (2.7)	8.9 (3.3)	1.8	0.108	0.651
III (PF2-PAK)	30.9 (6)	21 (9.3)	2.5	0.041 *	0.883
IV (PAK-PF3)	27.3 (10.7)	43.4 (15.2)	−4.8	0.002 *	1.701
V (PF3-end)	26.9 (5.6)	23 (6.7)	4	0.005 *	1.410
Time of peak angle (T%)					
ankle-sagittal	14.1 (2.9)	11.3 (2.7)	5.6	0.001 *	1.972
knee-sagittal	45.9 (6.5)	33.6 (9.4)	3.6	0.009 *	1.270
hip-sagittal	41.6 (6.6)	36.8 (6.9)	2	0.086	0.707

Notes: sagittal plane represents the flexion/extension (knee, hip) and dorsiflexion/plantar flexion (ankle). * indicates the significance level *p* < 0.05. Effect size (Cohen’s *d*), small: 0.2 ≤ *d* < 0.5, middle: 0.5 ≤ *d* < 0.8 and large: *d* > 0.8.

**Table 2:** Initial contact and peak angle, and range of motion (RoM) of ankle, knee and hip (mean ± SD).

	Pre-Fatigue	Post-Fatigue	*t*	*p*	Cohen’s *d*
Initial contact angle (degree)					
ankle-sagittal	10.4 (8.0)	6.4 (9.1)	1.4	0.212	0.486
knee-sagittal	13.1 (7.4)	7.9 (6.1)	4.2	0.004 *	1.476
hip-sagittal	46.1 (12.7)	42.2 (11.8)	2.5	0.044 *	0.868
ankle-frontal	11.1 (7.5)	13.4 (7.2)	−1.5	0.180	0.527
knee-horizontal	−19.1 (10.7)	−28.3 (19.1)	1.9	0.106	0.657
Peak angle (degree)					
ankle-sagittal (II)	−20.8 (5.7)	−18.2 (6.5)	−1.9	0.098	0.674
knee-sagittal	69.5 (8.8)	62.5 (8.8)	3.7	0.008 *	1.296
hip-sagittal	74.1 (11.9)	67.8 (12.8)	1.5	0.189	0.514
ankle-frontal (III and IV)	−3.4 (5.9)	−1.6 (4.5)	−1.1	0.337	1.395
knee-horizontal	1.4 (5.6)	−7 (17.3)	1.5	0.178	0.529
Range of motion (degree)					
ankle-sagittal	34.1 (8.1)	32 (8.2)	0.6	0.563	0.214
knee-sagittal	59.3 (7.3)	55.1 (6.3)	2.4	0.048 *	0.848
hip-sagittal	44.6 (10.4)	38.7 (10.8)	0.9	0.379	0.332
ankle-frontal	20.8 (9.8)	18.3 (3.5)	0.7	0.523	0.313
knee-horizontal	1.4 (5.6)	−6.9 (17.4)	1.5	0.180	0.526

Notes: sagittal plane represents the flexion/extension (knee, hip) and dorsiflexion/plantar flexion (ankle); frontal plane represents the eversion/inversion (ankle); horizontal plane represents the internal/external (knee). * indicates the significance level *p* < 0.05. Effect size (Cohen’s *d*), small: 0.2 ≤ *d* < 0.5, middle: 0.5 ≤ *d* < 0.8 and large: *d* > 0.8.

**Table 3:** Peak force (N/BW), peak moment (Nm/BW) and power (W/BW) (mean ± SD).

	Pre-Fatigue	Post-Fatigue	*t*	*p*	Cohen’s *d*
Peak force (N/BW)					
Initial	1.3 (0.1)	1.3 (0.2)	1.3	0.249	0.445
Secondary	1.6 (0.1)	1.5 (0.2)	1.7	0.142	0.584
Drive-off	1.3 (0.1)	1.2 (0.2)	1.6	0.158	0.558
Peak moment (Nm/BW)					
ankle-sagittal (I and II)	−0.6 (0.1)	−0.5 (0.1)	−2.1	0.072	0.679
ankle-sagittal (III and IV)	0.8 (0.1)	0.9 (0.3)	−0.9	0.402	0.341
knee-sagittal (I, II and III)	3 (0.7)	2.7 (0.5)	4.2	0.004 *	1.394
knee-sagittal (IV and V)	2.5 (0.6)	2 (0.5)	3.4	0.011 *	1.445
hip-sagittal (I, II andIII)	2.3 (0.5)	1.8 (0.6)	2.8	0.027 *	0.893
hip-sagittal (IV and V)	2.5 (0.4)	2.1 (0.5)	3.6	0.009 *	1.230
ankle-frontal (I)	0.1 (0.1)	0.1 (0.1)	−0.5	0.608	0.19
ankle-frontal (II)	−0.2 (0.2)	−0.1 (0.2)	−2.8	0.026 *	0.996
ankle-frontal (III)	−0.2 (0.2)	−0.1 (0.1)	−2.6	0.033 *	0.934
knee-horizontal (III and IV)	0.5 (0.5)	0.4 (0.4)	2.1	0.077	0.732
Peak power (W/BW)					
ankle-sagittal (I and II)	−6.1 (1.4)	−4.4 (1.8)	−1.9	0.104	0.661
knee-sagittal (I, II and III)	−17.2 (4.4)	−13.7 (3.1)	−3.6	0.009 *	1.268
knee-sagittal (IV and V)	10.9 (3.1)	6.1 (2.3)	6.6	0.000 *	2.325
hip-sagittal (I, IIandIII)	−6.9 (2.4)	−5.8 (2.5)	−1.6	0.15	0.571
hip-sagittal (IV and V)	4.4 (2.5)	2.2 (0.9)	3.3	0.014 *	1.157
knee-horizontal (I and II)	1.2 (0.8)	1.4 (0.8)	−0.8	0.474	0.288

Notes: sagittal plane represents the flexion/extension (knee, hip) and dorsiflexion/plantar flexion (ankle); frontal plane represents the eversion/inversion (ankle); horizontal plane represents the internal/external (knee). * indicates the significance level *p* < 0.05. Effect size (Cohen’s *d*), small: 0.2 ≤ *d* < 0.5, middle: 0.5 ≤ *d* < 0.8 and large: *d* > 0.8.

## Data Availability

Not applicable.
